# A comparison of task-based and job-based estimation of physical behavior compositions in grocery store workers

**DOI:** 10.1093/annweh/wxag056

**Published:** 2026-07-17

**Authors:** Svend Erik Mathiassen, Thomas Rudolfsson, Elin Vidlund

**Affiliations:** Department of Occupational Health, Psychology and Sports Sciences, University of Gävle, Kungsbäcksvägen 47, Gävle 801 76, Sweden; Department of Occupational Health, Psychology and Sports Sciences, University of Gävle, Kungsbäcksvägen 47, Gävle 801 76, Sweden; Department of Occupational Health, Psychology and Sports Sciences, University of Gävle, Kungsbäcksvägen 47, Gävle 801 76, Sweden

**Keywords:** occupational epidemiology, physical behaviors, task-based exposure assessment, Aitchison distance, compositional data analysis

## Abstract

**Background:**

Job exposure matrices (JEMs) are widely used in occupational epidemiology to estimate biomechanical exposures in a trade. However, JEMs are insensitive to variability among workers within the same job, potentially leading to less accurate exposures and increased uncertainty of exposure-outcome associations. Task-based exposure assessment, in which individual exposures are derived from task-specific exposures weighted by the time spent on each task, has been suggested to improve accuracy.

**Objective:**

To examine and compare the accuracy of task-based and job-based assessment of physical behaviors—sitting, standing, and moving—in grocery retail.

**Methods:**

The study was conducted in 2 Swedish medium-sized grocery stores. Accelerometry was used to measure “true” physical behaviors continuously over 3 full working days for all participating employees (*n* = 36; 16 women, 20 men). Job-based exposure estimates were derived by averaging sitting, standing, and moving across all workers, representing a JEM. Nine discrete tasks were defined in collaboration with employees and owners in the stores. For 35 of the workers, task timelines were videotaped for 4 h and linked to accelerometry to construct a gender-neutral (unisex) task exposure matrix (TEM). All 36 participants completed diaries for the 3 d, reporting time spent on each task, and task-based estimates of “true” exposures through the 3 d were calculated for each worker as the weighted average of task exposures based on the diary-reported task distribution. Physical behaviors were analyzed using procedures from compositional data analysis (CoDA), including describing and comparing the performance of job-based and task-based approaches using Aitchison distances to the measured, “true” exposures.

**Results:**

The prerequisites for successful task-based assessment were satisfied, including physical behavior contrasts between tasks and heterogeneity in task composition between workers. task-based estimates were on average closer to “true” exposures than job-based estimates, but the magnitude of improvement was modest (23%) and varied between women (35%) and men (12%). Thirty-three percent of the 36 “true” job exposures were *better* predicted by the job-based approach, and substantial variability in exposure persisted even within tasks.

**Conclusion:**

Task-based assessment offered only modest improvement in accuracy over job-based estimates and the cost-efficiency of the task-based approach compared to the less costly job-based strategy can be questioned. Task-based assessment may therefore be most useful when task information is needed for documentation or interventions, rather than solely for improving exposure estimation.

What's important about this paper? Using accelerometry, this study compared task-based estimates of sitting, standing, and moving among grocery store workers with estimates based on a Job Exposure Matrix (JEM). Even in this favorable setting, task-based estimates were on average only 23% more accurate, and 33% of workers were better estimated using the JEM. These findings lead to questioning the belief that task-based estimation of biomechanical exposures will always lead to more accurate estimates than job-based, and whether any possible gain in accuracy justifies the additional resources required.

## Introduction

Job exposure matrices (JEMs) addressing biomechanical exposures have gained increasing attention as tools for exposure assessment in occupational epidemiology (Dalbøge et al. 2016; Evanoff et al. 2019; Hanvold et al. 2019; Badarin et al. 2021; Korshøj et al. 2022; Solovieva et al. 2025). In a JEM, the same exposure estimate is assigned to all workers in a specific trade, typically the mean value of measurements obtained in a sample of workers. This approach enables exposure estimation for large populations at relatively low cost and has therefore been widely applied in epidemiologic studies, eg of work-related musculoskeletal disorders.

However, by construction, JEMs are insensitive to variability in exposure between workers within the same trade (Peters 2020). While this limitation may be acceptable in occupations where the exposure variability is small, it becomes problematic when workers within an occupation perform tasks with markedly different exposures to markedly different extents. In such cases, individual job exposures may deviate substantially from the average in the occupation, leading to considerable exposure misclassification.

As an alternative, task-based assessment of biomechanical exposures has been discussed (Benke et al. 2000; Heilskov-Hansen et al. 2014; Peters 2020). In task-based assessment, exposures are available at the level of work tasks in a “Task Exposure Matrix” (TEM) and estimates of individual job exposure are obtained by weighing the task-specific exposures in the TEM by the proportions of time each worker spends performing the tasks (Burdorf et al. 1997; Frost et al. 2002; Ditchen et al. 2015; Harris-Adamson et al. 2015; Garg et al. 2017). In principle, this approach allows for more accurate exposure estimation at the individual level than a JEM and thus offers opportunities for more refined analyses of both adverse and beneficial effects of occupational exposures.

Despite this theoretical advantage, few studies of biomechanical exposures have directly compared task-based and job-based exposure assessment within the same group of workers (Mathiassen et al. 2005; Svendsen et al. 2005). These previous investigations reported discouraging results, showing that task-based assessment provided little or no additional information beyond that obtained using a job-based approach. One possible explanation for the failure in these studies is that necessary conditions for successful task-based assessment were not met, let alone even checked a priori.

For task-based assessment to improve exposure estimation, workers within the same occupation must differ in their job exposures, and these differences must, to a satisfying extent, be explained by what tasks they do. Thus, the performance of a task-based approach depends on the proportion of between-worker exposure variability that can be explained by differences in the composition of tasks performed. While some degree of variability will always be present between and within individuals working in the same occupation (Wahlström et al. 2010, 2016; Brakenridge et al. 2016; Birk Jørgensen et al. 2019), a part of this variability may arise from factors unrelated to task composition, such as age, gender, or anthropometry (Johansson et al. 2020). Beyond this variability, 2 prerequisites must be fulfilled for a task-based assessment to be successful: (i) the job must comprise multiple tasks that occur with sufficient frequency and differ substantially in exposure; rare tasks will have negligible influence on overall exposure regardless of their specific exposure; (ii) workers must perform these tasks to different extents. Furthermore, it must be possible to determine both task exposures and task proportions with acceptable accuracy (Mathiassen et al. 2003).

Physical behaviors in grocery retail work, such as standing, sitting, and moving, represent a particularly attractive case for a successful task-based exposure assessment. First, physical behaviors are likely less influenced by individual characteristics than more complex biomechanical exposures such as working postures or muscle activity. Second, at visual inspection, the occupation comprises tasks characterized by distinctly different physical behaviors, fulfilling the criterion of exposure contrast between tasks. Third, task compositions may vary considerably between workers and across workdays, especially in retail environments of intermediate size, where staff are neither required to perform all tasks nor restricted to a single specialized role (Balogh et al. 2016). The performance of task-based assessment may, in this context, differ between women and men, as men may be more likely than women to switch between job tasks, and “male” tasks may have more variable exposures (Messing et al. 1998; Nordander et al. 1999; Calvet et al. 2012; Cherry et al. 2018).

Against this background, the present study examines the extent to which assessment of physical behaviors using a task-based approach provides more accurate job exposure estimates than a job-based approach in grocery retail work, and whether women and men differ in this aspect. Because physical behaviors are compositional (Chastin et al. 2015; Dumuid et al. 2018), analyses are conducted using principles from compositional data analysis (CoDA). Finally, the findings are discussed in relation to cost-efficiency, addressing whether any gain in exposure accuracy achieved through task-based assessment justifies the additional resources required to practice it.

## Materials and Methods

### Study setting and participants

The study was conducted in 2 medium-sized grocery stores in the suburbs of Stockholm, Sweden, employing at the time of the study 54 and 36 workers, 62% and 60% of whom were women. The stores belonged to the same corporate group, and they were selected by convenience after a recruitment campaign covering 22 stores in the greater Stockholm area. Data collection took place April–May 2018 in the first store, and March–May 2019 in the second. All eventual participants signed informed consent prior to participating, and the study was approved by the Regional Ethical Review Board in Uppsala, Sweden (DNr. 2017/404).

### Baseline questionnaire

We introduced the study to workers in the stores at staff meetings. After the meeting all employees working during the study period received an email with a link to a web-based questionnaire. The questionnaire covered demographics such as sex, age, educational level, and number of years within the occupation (Mathiassen et al. 2020). Workers preferring to answer a pen-and-paper version of the questionnaire were allowed to do so. Later, we approached individual workers while working in the store, asking them to participate in measurements of physical behaviors, while also reminding them of filling in the questionnaire.

### Tasks

In collaboration with store owners and employees, we established a complete list of 9 work tasks to ensure that task definitions were meaningful and recognizable to workers. Task definitions were consistent across the 2 stores, and corresponded to the system used internally for planning, managing, and monitoring work. *Checkout* comprised work in the checkout counter as well as assistance to customers in a self-service checkout. *Colonial*, *FruitVeg(etables)*, *Dairy* and *Bread* stood for work in each of 4 sections in the store, designated to handle colonial products, fruits and vegetables, dairy products and frozen goods, and bakery products, respectively. In all cases, work consisted of receiving or (in *Bread*) preparing goods and stocking them on shelves or in specific counters in the store. In *Fresh*, the worker serviced customers at a separate counter offering perishable products, such as fresh meat, fish, and salads, and in *Post,* customers were serviced regarding delivery and pick-up of packages and letters on commission from different Swedish postal services. *Admin(istration)* mainly implied paperwork or work at a computer to manage the sections in the store, or to communicate with other stores and the corporate group. Finally, *Break* was time off work, mainly spent in backstage premises in the stores.

### Measurement of physical behaviors

We measured physical behaviors at work using thigh-worn accelerometers (Actigraph GT3X+, Ametris, Pensacola, Florida, USA) continuously during 3 complete working days. A researcher met the worker at the beginning of a work shift and attached the accelerometer to the right thigh with the x-axis pointing towards the foot, fixing it by a double-coated adhesive tape and covering it with a plastic adhesive film (Opsite Flexifix, Smith & Nephew, London, UK). The accelerometer was initialized using the OmGui software (V.1.0.0.30; Open Movement, Newcastle University, UK) and collected data at 25 Hz. We processed accelerometer data using the Acti4 software (National Research Center for the Working Environment, Copenhagen, Denmark), which identifies periods of sitting, standing, walking, running, cycling, and climbing stairs with high sensitivity and specificity (Skotte et al. 2014; Stemland et al. 2015). Time at work used for walking, running, cycling, and climbing stairs was later merged into one behavior category, “moving”.

### Compositional data analysis (CoDA)

Physical behaviors (*in casu* sitting, standing, moving) are compositional as they represent parts of a whole workday. We therefore processed data using principles of compositional data analysis (CoDA; Dumuid et al. 2020; Gupta et al. 2020). Prior to analysis, we checked all workers for zero time (which is not allowed in CoDA) occurring in any behavior in tasks that the worker performed. Zeros occurred in *FruitVeg* and *Post* for one worker each, and in *Break* for 5 workers, and were replaced by 0.01%time under the assumption that these zeros resulted from limited data. At replacement, we reduced time spent on other behaviors by an equivalent amount so that total time was preserved at 100% (Lund Rasmussen et al. 2020).

### ‘True’ job exposures and job-based exposure estimation

For each individual worker, we determined the total proportion of working time spent sitting, standing, and moving for each workday, and averaged across the 3 measurement days to get an estimate of the “true” occupational job exposure. The mean value of these “true” job exposures across all workers corresponds to a Job Exposure Matrix (JEM), and we assigned it to each worker as a gender-neutral (unisex) job-based exposure estimate. We also assessed the variability between workers in “true” job exposure using a dispersion metric based on the index suggested by Aitchison for assessing the distance between 2 (three-part) compositions, *A* and *B* (equation (1) in Aitchison et al. 2000):


(1) $${\left[\overset{3}{\underset{i=1}{\sum }}\;{\left\{\ln\frac{x_{i}A}{G(A)}-\;\ln\frac{x_{i}B}{G(B)}\right\}}^{2}\right]}^{\frac{1}{2}}$$

where *x_i_A* and *x_i_B* denote the *i*th parts (coordinates) of composition *A* and *B*, and *G(A)* and *G(B)* are the geometric means of the 2 compositions, ie (*x_1_A·x_2_A·x_3_A*)^1/3^ and (*x_1_B·x_2_B·x_3_B*)^1/3^. When this equation is used for calculating the dispersion of Aitchison distances of individual behavior compositions, *A*, to their common mean, composition *B* refers to the (arithmetic) mean composition, and distances of individual compositions to the mean are averaged. This “Aitchison Dispersion Index” (ADI) represents a compositional analogue to the standard deviation (SD).

### Task exposure matrix

We videotaped workers for 4 h on the second day of accelerometry measurements. Off-line, we examined the videotapes to identify sequences in each of the 9 tasks (if present), which were then synchronized with the corresponding accelerometer data. These data were used to construct a gender-neutral task exposure matrix (TEM), consisting of average physical behavior compositions in each task, with ADIs as measures of within-task between-worker variability. We also calculated the variability between tasks in terms of an ADI based on the dispersion of the mean exposures of the 9 tasks.

### Task proportions and task-based exposure estimation

All workers participating in accelerometry were asked to complete diaries for the same 3 d during which measurements were taken. The diary requested the workers to note when they began working every day and when they stopped, and the points in time when they changed between any of the 9 predefined tasks. Participants were included in further analyses only if they performed their regular in-store duties during all 3 d according to the diary. The diaries provided the basis for calculating the percentage of working time spent in each task for each day and worker. These daily task distributions were averaged to give the worker's task distribution for all 3 d together. Arithmetic mean values of time proportions in the 9 tasks were calculated both across all workers, representing the overall occurrence of the tasks in the stores, and across only those workers who actually performed the tasks, as a metric indicating the extent of task specialization among workers. In the latter case, variability between workers was assessed using ADIs, yet with only 2 parts in the composition (ie *i* = 2 in equation (1)); ie time spent on the task, and time spent on any other task.

We then calculated task-based job exposure estimates for each individual worker as a weighted average of task exposures from the TEM, with weights equal to the overall percentage of working time spent in each task for that worker. Finally, we calculated arithmetic means of the task-based estimates (with ADIs), both for all workers and stratified by gender.

### Performance of task-based and job-based exposure estimation

We evaluated the performance of task-based and job-based exposure procedures by comparing, for each worker, the distances from both job exposure estimates to the “true” job exposure for the same individual, as derived from the full 3-d accelerometry recording. Distances were calculated using equation (1), but now with composition *A* representing either the task-based or the job-based estimate and composition *B* being the individual worker's “true” exposure composition.

These individual Aitchison Distances to the “truth” were summarized in terms of mean distances (with SDs) across all participants for the task-based and job-based strategies, and the overall performance of the task-based strategy was expressed as the ratio between the task-based and job-based mean distances. Analyses were conducted for the total sample and separately for men and women.

### Sensitivity analysis

Three participants (2 women, 1 man) reporting *check-out* were observed to work mainly in the self-service checkout stations, where staff assisted customers and surveilled transactions. These workers were mostly standing, as opposed to those working in the check-out counters, who were preferentially sitting. Thus, *check-out* was inconsistent in terms of physical behavior. To assess the influence of this source of uncertainty on task-based estimation, we performed a sensitivity analysis in which these 3 participants were excluded.

## Results

### Participants

Out of the, in total, 90 workers employed in the 2 stores, 57 (63%) answered the baseline questionnaire (Table 1). Thirty-one of these (16 women, 15 men) also participated in the 3-d accelerometry measurements (Table 1). All 16 women participating in the 3-d accelerometer measurements also answered the questionnaire, while 5 men signed up for accelerometry without answering the questionnaire. Thus, the sample for examining the performance of task-based assessment consisted of 16 women and 20 men. One woman declined to be followed by an observer for 4 h, resulting in 15 women and 20 men providing the basis for the TEM.

**Table 1:** Demographics of the study population. Data shown for workers answering the baseline questionnaire as well as for workers also participating in accelerometry measurements.

	Baseline			Baseline and accelerometry		
	All (*n* = 57)	Women (*n* = 35)	Men, (*n* = 22)	All (*n* = 31)	Women (*n* = 16)	Men (*n* = 15)
Age, yrs [mean (SD)]	29 (11.0)^b^	30 (11.4)^b^	29 (10.7)	31 (11.1)^a^	34 (12.6)^a^	27 (8.4)
Height, m [mean, SD]	1.72 (9.4)^a^	1.67 (6.3)	1.81 (6.5)^a^	1.73 (9.8)	1.67 (7.2)	1.80 (7.0)
Weight, kg [mean, SD]	70.1 (13.5)^c^	62.2 (7.8)^b^	83.0 (10.7)^b^	72.2 (15.3)^a^	61.4 (9.8)^a^	83.0 (11.8)
BMI^d^, kg·m^−2^ [mean, SD]	23.6 (3.2)^c^	22.4 (2.1)^b^	25.5 (3.8)^b^	23.9 (3.8)^a^	22.2 (2.7)^a^	25.6 (4.0)
Country of birth [*n* (%)]						
Sweden	54 (94.7)	33 (94.3)	21 (95.5)	29 (93.5)	15 (93.8)	14 (93.3)
Outside Sweden	3 (5.3)	2 (5.7)	1 (4.5)	2 (6.5)	1 (6.2)	1 (6.7)
Job title [*n* (%)]						
Manager^e^	17 (29.8)	9 (25.7)	8 (36.4)	14 (45.2)	7 (43.8)	8 (53.3)
Shop assistant	40 (70.2)	26 (74.3)	14 (63.6)	17 (54.8)	9 (56.3)	7 (46.7)
Employment status [*n* (%)]						
Full-time	19 (33.3)	10 (28.6)	9 (40.9)	14 (45.2)	7 (43.8)	7 (46.7)
Part-time	38 (66.7)	25 (71.4)	13 (59.1)	17 (54.8)	9 (56.2)	8 (53.3)
Employment tenure, yrs [mean, SD]						
In any store	8.3 (9.9)	6.7 (9.7)	10.4 (10.0)	8.3 (10.1)	9.1 (13.4)	7.5 (6.6)
In the current store	5.7 (7.2)	4.5 (7.5)	7.4 (6.7)	6.1 (7.4)	6.7 (10.3)	5.5 (3.8)
Current pain^f^ [*n* (%)]						
Neck	30 (53.6)^a^	20 (58.8)^a^	10 (45.5)	16 (51.6)	9 (56.3)	7 (46.7)
Shoulders	33 (57.9)	25 (71.4)	8 (36.4)	15 (48.4)	11 (68.8)	5 (33.3)
Low back	31 (54.4)	22 (62.9)	9 (40.9)	15 (48.4)	10 (62.5)	5 (33.3)

^a^Data missing for 1 worker

^b^Data missing for 2 workers

^c^Data missing for 4 workers

^d^BMI = weight/height^2^

^e^Workers were classified as “managers” if they had any administrative duties in sections of the stores or the entire store

^f^Answers to the question “Have you experienced any problems (pain, aches, discomfort) in the [neck/shoulders/low back] at any time during the past 3 mo?”

Male workers were to a larger extent full-time employed with managerial tasks than females, and reported pain in the neck, shoulders and low back to a less extent (Table 1). Workers participating in accelerometry were, in general, more likely to be managers and have full-time employment than those answering the baseline questionnaire (Table 1).

### ‘True’ job exposures and job exposure matrix

**Figure 1:**
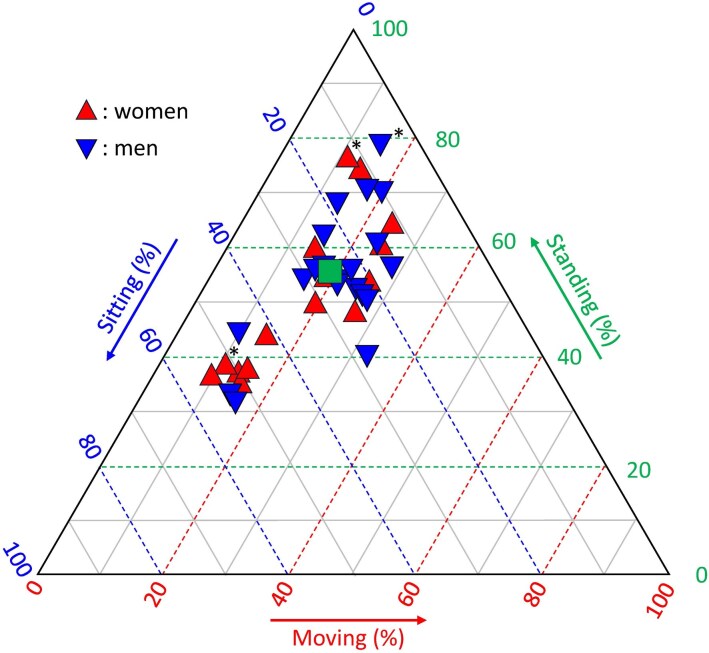
“True” job exposure compositions according to the 3-d accelerometry measurements for women (red triangles, *n* = 16) and men (blue inverted triangles, *n* = 20). Green square: arithmetic mean across all workers, ie the Job Exposure Matrix composition. *: the 3 workers excluded in the sensitivity analysis.

The arithmetic mean composition of sitting, standing, and moving during the 3 working days with accelerometry for all 36 participants, ie the group mean of “true” job exposures used as the gender-neutral JEM, was 27.4%/53.8%/18.7% sit/stand/move. We observed a difference between women (mean composition 30.6%/51.6%/17.8%) and men (24.9%/55.6%/19.5%). The “true” job exposures are illustrated in Fig. 1 and detailed in Table S1. Geometric “true” job exposure means, scaled to a total of 100%time, were 25.5%/55.4%/19.0% (sit/stand/move) for all participants, 28.6%/53.3%/18.1% for women and 23.3%/57.1%/19.7% for men. ADIs, measuring the between-worker variability in “true” job exposure, were 0.526 (all), 0.552 (women) and 0.505 (men).

### Tasks, task proportions and task exposure matrix

All 9 predefined work tasks were reported during the 3-d measurement period (Table 2). Data for workers *actually performing* each task indicated some specialization (ie large average time proportions in the task, compared to the overall mean across all workers), in particular for *Dairy*, *Bread* and *Fresh*. The occurrence of the 8 productive tasks (ie disregarding breaks) differed considerably between men and women (Table 2; detailed in Table S2). The data suggested that women were more specialized than men on doing only few tasks. Out of the 16 female workers, 9 (56%) performed a single task for more than 90% of their productive time, while only 8 of the 20 men (40%) were equally specialized.

**Table 2:** Task distributions. Percentage of time spent on each of the nine tasks according to the three-day diary across all workers (*n*=36), and across workers actually doing the task. Arithmetic mean values and between-worker variability, measured using the Aitchison Dispersion Index (ADI). For workers doing the task, data are provided for the entire sample and for women and men separately.

	All workers	Workers doing the task								
		All			Women			Men		
	Time in task (%)^a^	*n*	Time in task (%)	ADI	*n*	Time in task (%)	ADI	*n*	Time in task (%)	ADI
Checkout	22.6	15	54.4	0.99	6	67.2	0.77	9	45.8	0.94
Colonial	21.5	21	36.9	1.11	9	35.5	1.20	12	37.9	1.04
FruitVeg	11.8	10	42.4	1.50	4	44.8	1.61	6	40.9	1.42
Dairy	6.4	6	38.3	0.75	0	…	…	6	38.3	0.75
Bread	4.3	3	52.1	0.65	3	52.1	0.65	0	…	…
Fresh	8.4	7	43.3	1.32	2	46.7	1.66	5	41.9	1.19
Post	7.3	8	32.6	0.89	3	43.0	1.12	5	26.4	0.85
Admin	5.6	18	11.2	0.70	6	19.9	0.95	12	6.9	0.52
Break	12.1	36	12.1	0.19	16	12.5	0.19	20	11.7	0.19

^a^Percentages show the time spent on each task in the overall production in the stores, ie the sum is 100%. ADI was not possible to calculate due to an extensive occurrence of zeros in several tasks

Descriptive data by task on physical behaviors during the 4-h observation period, ie the TEM, are presented in Table 3 (detailed in Table S3). These results demonstrate substantial differences across tasks, with *Checkout* and *Admin* being dominated by sitting, while *Colonial*, *FruitVeg*, *Dairy*, *Bread*, *Fresh* and *Post* were mainly performed standing. The between-task ADI was 1.12 (not shown in Table 3). We also found varying degrees of exposure homogeneity within tasks, as measured by the within-task between-worker ADI (Table 3). For instance, variability was considerably larger in *Post* than in *Admin*, likely because physical behaviors in *Admin* were more stereotyped—mainly consisting in sitting—than in *Post*, which included both standing and sitting operations in proportions varying by worker.

**Table 3:** The task exposure matrix. Compositions of the three physical behaviors (sitting, standing, moving) in each of the nine tasks for those observed to perform the task during the four hours of surveilled data collection. Number of workers (*n*), available time of accelerometry (Measurement time), arithmetic mean values of percentage time in sitting, standing and moving, and between-worker variability measured using the Aitchison Dispersion Index (ADI). Data are provided for the entire sample of workers, and for women and men separately.

	All						Women						Men					
	*n*	Time measured (min)	Task Exposure Matrix (% time)			ADI	*n*	Time measured (min)	Time in task (%)			ADI^a^	*n*	Time measured (min)	Time in task (%)			ADI^a^
			sit	stand	move				sit	stand	move				sit	stand	move	
Checkout	13	1,761.4	58.0	33.7	8.3	2.27	6	907.2	50.7	39.6	9.7	2.65	7	854.3	64.2	28.7	7.1	1.92
Colonial	19	2,111.1	14.7	62.5	22.8	0.53	8	942.0	17.7	60.8	21.4	0.53	11	1,169.1	12.5	63.7	23.8	0.48
FruitVeg	4	701.8	10.4	71.4	18.2	1.72	1	216.3	7.1	73.7	19.2	…	3	485.5	11.4	70.7	17.9	2.15
Dairy	6	797.0	10.0	71.1	18.9	0.75	0	…	…	…	…	…	6	797.0	10.0	71.1	18.9	0.75
Bread	2	284.3	7.7	70.3	21.9	0.33	2	284.3	7.7	70.3	21.9	0.33	0	…	…	…	…	…
Fresh	6	868.2	5.3	72.3	22.4	0.59	1	224.9	1.2	83.7	15.1	…	5	643.3	6.2	70.0	23.8	0.50
Post	4	277.4	10.1	68.3	21.6	2.54	1	29.7	37.1	42.1	20.8	…	3	247.8	1.1	77.1	21.8	1.71
Admin	6	827.8	80.2	15.7	4.1	1.05	2	169.9	71.6	25.9	2.4	1.15	4	657.9	84.6	10.5	4.9	0.84
Break	29	726.6	43.6	34.0	22.5	2.08	10	243.5	41.3	31.5	27.2	1.79	19	483.2	44.7	35.3	20.0	2.22

^a^Reported only if ≥2 participants.

### Performance of task-based vs. job-based exposure estimation

**Figure 2:**
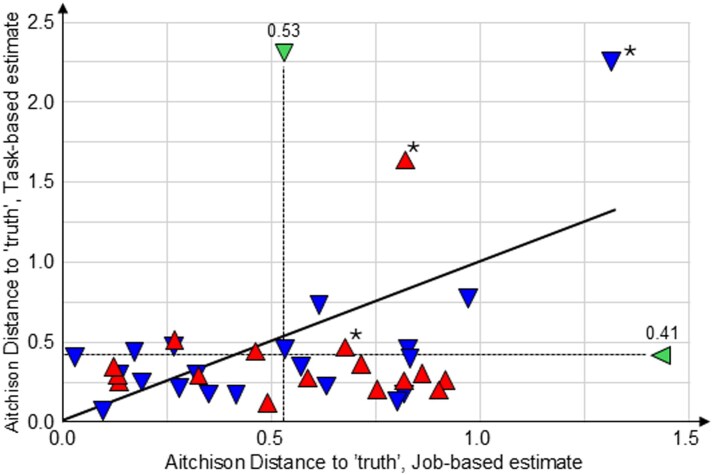
Performance of job-based (x-axis) and task-based (y-axis) estimates of physical behaviors in terms of Aitchison Distances to “true” job exposures for women (red triangles, *n* = 16) and men (blue inverted triangles, *n* = 20). The figure includes the line of identity (black); for workers above the line, estimates based on the Job Exposure Matrix (JEM) were the more accurate, while for workers below the line, the approach utilizing the Task Exposure Matrix (TEM) was the better. Dashed lines with green triangles indicate the mean Aitchison Distances of job-based (0.53) and task-based (0.41) estimates. * the 3 workers excluded in the sensitivity analysis.

Overall, the task-based approach resulted in a smaller mean Aitchison Distance to the “true” exposure, 0.41, than the job-based approach, 0.53 (Fig. 2; detailed in Table S1). Thus, the average Aitchison Distance to the “truth” in the task-based approach was 77.4% of that in the job-based approach, with markedly better task-based performance among women (65.2%, Aitchison Distances 0.36 (task-based) and 0.55 (job-based)) than among men (88.1%, Aitchison Distances 0.45 and 0.51). Figure 2 also illustrates the dispersion among individuals of Aitchison Distances to the “truth” for both the job-based and task-based approach. For 24 of the workers (67%) the task-based estimate was, indeed, closer to the “truth” than the job-based estimate, but for 12 workers (33%) task-based estimates were *less* accurate than job-based estimates. These percentages were similar for men and women (Fig. 2; Table S1). Visual inspection of Fig. 2 suggests that workers with larger job-based distances tended to benefit more from the task-based approach, whereas task-based estimation provided limited or no improvement, and in many cases even a deterioration, for workers whose job-based estimates were already close to the “true” exposure.

### Sensitivity analysis

The 3 workers performing *checkout* predominantly in a standing posture were confirmed to almost never sit down in this task (Table S3). Excluding them changed the average task exposure in *checkout* from 58.0%/33.7%/8.3% (sit/stand/move; Table 3) to 75.3%/20.0%/4.7%. The ADI (between-subject variability) in *checkout* decreased from 2.27 to 1.01, verifying that workers now experienced more homogeneous exposures (predominantly sitting). However, the exclusion had only limited effect on group-level exposure estimates and on the comparative performance of the job-based and task-based approaches: the performance of task-based compared to job-based assessment changed from 77.4% to 74.1% (women 65.2% to 58.2%; men 88.1% to 87.3%).

## Discussion

### Performance of task-based versus job-based exposure assessment

The central aim of the present study was to evaluate the relative performance of task-based exposure assessment under conditions where it could reasonably be expected to perform well. The descriptive results indicate that these preconditions were largely fulfilled. First, the task-specific exposure summaries showed clear differences in physical behavior composition between tasks, indicating a meaningful exposure contrast (Table 3, Table S3). Second, the distribution of task proportions across workers demonstrated substantial heterogeneity in task composition among individuals, both overall and within gender strata (Table 2, Table S2). Together, these findings suggest that grocery retail satisfies the 2 fundamental requirements for task-based assessment identified in the introduction: exposure contrast between tasks and differences between workers in task composition.

Despite these favorable conditions, the improvement in exposure estimation achieved by the task-based approach compared with the job-based approach was modest. On average, task-based estimates were closer to the “true” exposures than job-based estimates by 23% but for a substantial proportion of workers, the job-based estimate was actually closer to the “truth”. These findings are consistent with previous comparisons of task-based and job-based approaches, which have generally reported limited, if any, added accuracy with task-based assessment (Mathiassen et al. 2005; Svendsen et al. 2005). Svendsen et al. addressed arm elevation >90° in machinists, car mechanics and house painters, and found marginal gains in accuracy when adding task-based individuals to a database of workers for whom “true” exposure was already available. Mathiassen et al. (2005) studied upper trapezius exposure among cleaners and office workers and also found job-based and task-based estimates of exposure to be equivalent, in spite of clear exposure differences between tasks, particularly among the cleaners. In both of these studies, task exposures were based on direct measurements while task proportions were obtained by diaries (Svendsen et al. 2005) or observation (Mathiassen et al. 2005).

The present results extend this earlier work by demonstrating that even under conditions that appeared optimal for task-based assessment, a considerable proportion of between-worker variability in job exposure remained unexplained. Even within the same task, exposure variability between workers could be substantial (Table 3). This observation aligns with studies in office environments, where workers performing nominally similar tasks still exhibit considerable variability in physical behaviors (eg Healy et al. 2016; Huysmans et al. 2019; Johansson et al. 2020). This residual variability likely reflects individual work style, organizational factors, and contextual influences, and its considerable magnitude suggests that the potential to improve exposure estimation accuracy through a task-based approach may be lower than often assumed.

### Physical behaviors in grocery retail

The overall composition of sitting, standing, and moving observed in grocery retail in the present study was similar to that reported in other predominantly standing and ambulatory occupations such as eldercare (Lögdal et al. 2026) and cleaning (Birk Jørgensen et al. 2019). Compared with office-based work (Brakenridge et al. 2016; Renaud et al. 2020), grocery retail involved substantially more standing and moving, with sitting consequently constituting a smaller proportion of the workday. Moving occurred to a less extent in the stores than in occupations with extensive physical activity such as refuse collection (Hallman et al. 2015) and order picking in distribution centers (Lavender et al. 2025). Other directly measured biomechanical exposures in retail have been reported, including muscle activity and upper extremity postures (Rissén et al. 2000; Balogh et al. 2016; Skals et al. 2021a) and joint reaction forces in the knees, spine, and shoulders (Skals et al. 2021b), but exposures other than physical behaviors were not examined in the present study.

### Task classification

Task classification systems developed explicitly for use in task-based exposure assessment aim to balance contrasts in exposure between tasks with usability for the workers or researchers supposed to understand and identify tasks, in many cases during on-going work (Svendsen et al. 2005). This creates an inherent trade-off between statistical performance and practical feasibility. Increasing the granularity of task classification may be counterproductive, first since the additional uncertainty introduced in estimating task proportions may outweigh gains in exposure accuracy, second because the TEM will be based on less observations in each task and therefore be more uncertain. On the other hand, overly coarse task definitions may obscure meaningful exposure differences. A useful compromise may, however, be difficult to find, and it may only be possible for certain exposures and in some occupations. In the present study, we identified a source of inaccuracy in the *checkout* task exposure: the task encompassed both work at the checkout counter, which was predominantly sitting, and assistance to customers at self-service checkout counters, which was mainly standing. While correcting for this inconsistency in the present case had only a marginal effect on the performance of task-based exposure assessment, it motivates consideration to whether modifying a default task classification can improve the accuracy of task-based estimates while remaining readily understandable to workers (eg Mathiassen et al. 2005; Svendsen et al. 2005; Healy et al. 2016; Huysmans et al. 2019; Johansson et al. 2020).

### Task exposures and task proportions

The limited number of participants in the present study precluded gender-specific task exposure estimates, since either women or men were too few in several of the 9 tasks to justify a stratification (cf. Table 3). Task exposure databases including data for both women and men exist for other exposures in other trades (eg Heilskov-Hansen et al. 2014), but their usefulness for task-based exposure assessment has not been explicitly evaluated.

We assessed task proportions using self-reported diaries. However, evidence suggests that errors in self-reported task proportions can be substantial and influenced by organizational and contextual factors (Hunting et al. 2010; Pulido et al. 2018; Kersten and Fethke 2019), and that they increase if tasks are rare (Unge et al. 2005). Errors in task proportions translate directly into inaccurate task-based exposure estimates and likely reduce the performance of task-based assessment (Mathiassen et al. 2003). In some occupations, administrative or operational data are available, describing when and for how long individual workers perform specific tasks. This could serve as an alternative to self-reports (Svendsen et al. 2005) and may even be useful for predicting task exposures (van der Beek et al. 2013). The potential of using administrative data for exposure estimation in different trades warrants further investigation, and opportunities will likely increase as part of the digital development (Howard 2019).

A source of error, both in task exposures and task proportions, is periods of transition between tasks, for instance when walking between different areas in the grocery store. These effects may be particularly pronounced for workers who frequently shift between tasks, even if they are, for instance, specifically instructed to note the point in time when they begin another task. Frequent shifts are likely to introduce greater dilution of task exposures, both by increasing periods that are not attributable to any task and also by reducing the accuracy of task proportions because workers will likely not note every shift if the frequency is high.

### Task-based exposure assessment stratified by gender

Some earlier evidence indicates that men and women often have similar exposures when performing the same tasks (Heilskov-Hansen et al. 2014), and this may particularly apply to exposures such as physical behaviors that are less dependent on body dimensions or motor control than, eg postures and muscle activity. However, differences in physical behavior between genders within tasks may have been present in the stores, as exemplified by *Colonial* and *Break* (Table 3). However, our data did not allow a gender-stratified TEM. Using a gender-neutral TEM also facilitates interpretation of gendered differences in task-based job exposure estimates, since such differences can only be caused by a differential task allocation between genders. In the present study, average task-based exposure estimates differed between women and men (Table S1), confirming that men and women differed to a notable extent in the tasks they performed. As an example, the bread counter was staffed only by women, whereas dairy handling was performed only by men. Inequality regimes in the stores are discussed in more detail in another paper based on interviews with the workers (Olofsdotter et al. 2023).

### Cost-efficiency

Statistical performance alone cannot justify selecting task-based assessment over job-based assessment; costs associated with each of the two approaches must also be considered. Compared with the job-based approach, task-based assessment requires additional resources from researchers and study participants both to construct the task exposure database and to determine individual task proportions. To obtain a gain in cost-efficiency, the relative increase in accuracy with the task-based approach should be larger than the relative increase in cost (Rezagholi et al. 2012). In the present study, the improvement in accuracy over job-based assessment was modest, ie 23% for the whole sample, which raises the question of whether a gain in efficiency of this size justifies the additional cost associated with our task-based approach.

From a broader cost-efficiency perspective, task-based assessment should be evaluated relative to the full spectrum of available job-based strategies, ranging from a JEM based on expert ratings to simple means of self-reported job exposures to full-day individual measurements. We encourage more research into the comparative cost-efficiency of feasible strategies, as well as of different data sampling schemes within a particular strategy (Rezagholi et al. 2012; Mathiassen et al. 2013), and even into the performance of different measurement methods for retrieving the same data (Trask et al. 2014; Mathiassen et al. 2023). Notably, technological advances are rapidly changing the cost structure of several alternatives. While full-shift measurement of all workers will likely become increasingly cost-efficient for exposures such as physical behaviors that can be accessed through accelerometry (Crowley et al. 2022), task-based assessment may still be an option for exposures that are costly to assess, such as electromyography. Notably, task information may be of interest for other purposes than job exposure estimation, for example identification of tasks with particular needs for ergonomics interventions (eg Fethke et al. 2015; Douphrate et al. 2017), or reorganization of tasks to obtain better jobs for the workers, as in a job rotation (eg Jackson et al. 2026).

### Strengths and limitations

The present study was small, consisting of 36 participants: 16 women and 20 men. The limited number of women and men and the resulting occurrence of zeroes in some task categories led us to construct a gender-neutral TEM rather than a gender-stratified one. Summary statistics for the task-based estimates of the whole group, *n* = 36, may, however, be more stable than estimates from stratified samples of smaller size, such as in Svendsen et al. (2005) with 23–26 participants in each of 3 occupational groups, and Mathiassen et al. (2005) examining 23–24 participants in 2 groups. Also, the gender-neutral TEM facilitated examination of gendered differences in task-based job exposure estimates, as noted above.

Both the JEM and the TEM were constructed using data from the same population in which performance was evaluated, which may inflate performance for each of the approaches (Mathiassen et al. 2005; Coenen et al. 2020). External validation was not possible, and performance would likely be lower for both approaches had the present JEM or TEM been applied in other stores. The relationship between job-based and task-based estimates may, however, not be affected to any major extent. Moreover, exposure estimates were evaluated over the same days for which task diaries were available, providing strong internal validity but offering limited insight into exposure variability and its effects on statistical performance over longer periods, such as weeks or months.

Short-term day-to-day variability in exposure was not examined, despite task-based assessment being well suited for such analyses in contrast to the job-based approach. The task-based approach used by us also assumed independence between task proportions and task exposures, and we did not examine whether this assumption was correct. Any possible dependence may, however, have effects both on the accuracy of task-based estimation and on the relationship between task-based and job-based exposure estimation.

We used self-reported diaries to assess task proportions. This is a limitation, since self-reported time in tasks is likely inaccurate to some extent (Pulido et al. 2018). However, we found diaries to be an attractive and feasible approach in the present case, compared to, eg observing 36 participants continuously for 3 d each, let alone that even an observer may find it challenging to accurately identify tasks (Unge et al. 2005). We encourage further research into the cost-efficiency of investing resources at extensive observations to get estimates of task proportions that are, likely, more accurate than those obtained by comparatively cheap self-reports.

The present study only reported exposure to physical behaviors, ie sitting, standing, and moving. This may be considered a limitation, given that several other exposures may also be relevant to the development of disorders among retail workers (Anton and Weeks 2016), including, eg postures and loads of the low back and upper extremity (Skals et al. 2021a), and weight and frequency of lifting operations (Bláfoss et al. 2024). However, rather than aiming to provide a comprehensive selection of exposures relevant to grocery retail, the primary purpose of the present study was to compare task-based and job-based exposure assessment for an exposure considered particularly promising for obtaining good relative efficiency of task-based assessment, ie physical behaviors.

Finally, methodological challenges related to compositional data analysis deserve consideration. Descriptive statistics based on arithmetic means may be more useful than scaled geometric means, as prescribed by standard CoDA procedures (Chastin et al. 2015; Dumuid et al. 2018). For instance, geometric means were not of any use when documenting the mean time spent on individual tasks across all workers in the stores (ie in the overall production), due to genuine zeros in all behaviors for those workers not performing the tasks. In the present study, we used metrics based on Aitchison Distances (Aitchison et al. 2000) to quantify dispersion between participants and agreement between task-based and job-based exposure assessment strategies, as opposed to using more intricate compositional metrics (eg Lund Rasmussen et al. 2026). However, the properties of the Aitchison Dispersion Index (ADI) as a measure of between-subject variability, and of Aitchison Distances as a metric of agreement, need further examination in research and practice, including, eg the consequences of different zero replacement techniques (Lund Rasmussen et al. 2020).

## Conclusions

Grocery retail represents a favorable context for task-based exposure assessment, with clear exposure contrasts between tasks and heterogeneous task compositions between workers. Even under these conditions, however, task-based assessment provided only modest improvements over a job-based approach. This suggests that task-based exposure assessment is unlikely to be broadly beneficial across occupations. Future research should clarify the conditions and purposes for which a task-based approach justifies the additional resource investment compared with a job-based approach.

## Supplementary Material

wxag056_Supplementary_Data

## Data Availability

The data used are available on reasonable request.
