# Supplementary material for: A comparison of task-based and job-based estimation of physical behavior compositions in grocery store workers
Source: Ann Work Expo Health. 2026 Jul 17;70(6):wxag056. doi: 10.1093/annweh/wxag056 (PMC13378765; doi:10.1093/annweh/wxag056)
Supplement: wxag056_Supplementary_Data [file wxag056_supplementary_data.pdf]

Supplementary Material

A comparison of task-based and job-based estimation of physical behavior compositions in grocery store workers

Svend Erik Mathiassen

Thomas Rudolfsson

Elin Vidlund

Department of Occupational Health, Psychology and Sports Sciences, University of Gävle, Kungsbäcksvägen 47, 801 76 Gävle, Sweden

Supplementary table S1.

"True" compositions of sittting, standing and moving in the job for each worker as well as Job-based and Task-based estimates with Aitchison distances to the "truth".

Blue cells mark data for the three workers excluded in the sensitivity analysis

	"True" Job exposures according to three-day measurements			Job-based estimates of the "truth" using the JEM			Task-based estimates of the "truth" using the TEM			Aitchison distances to the "truth"	
	%Sitting	%Standing	%Moving	%Sitting	%Standing	%Moving	%Sitting	%Standing	%Moving	Job-based	Task-based
**Women**											
	11.3	71.4	17.3	27.4	53.8	18.7	10.1	67.4	22.3	0.849	0.276
	24.7	57.8	17.5	27.4	53.8	18.7	19.7	62.6	17.5	0.130	0.224
	28.5	46.3	25.3	27.4	53.8	18.7	24.8	54.3	20.8	0.319	0.268
	24.7	55.4	19.9	27.4	53.8	18.7	19.2	59.8	20.9	0.124	0.257
	47.6	38.0	14.4	27.4	53.8	18.7	55.7	33.7	10.5	0.704	0.334
	49.1	36.1	14.8	27.4	53.8	18.7	46.7	40.2	13,0	0.743	0.172
	40.7	43.7	15.6	27.4	53.8	18.7	37.8	45.9	16.1	0.482	0.094
	46.5	38.7	14.8	27.4	53.8	18.7	55.7	34.6	9.5	0.666	0.440
	20.0	54.2	25.9	27.4	53.8	18.7	13.4	64.8	21.6	0.453	0.411
	25.2	60.3	14.5	27.4	53.8	18.7	20.4	56.7	22.7	0.261	0.485
	49.4	38.8	11.8	27.4	53.8	18.7	56.5	32.1	11.2	0.809	0.228
	11.6	62.9	25.6	27.4	53.8	18.7	13.8	64.1	22,0	0.903	0.233
	51.8	37.1	11.1	27.4	53.8	18.7	46.4	41.1	12.3	0.890	0.174
	29.8	50.2	20.0	27.4	53.8	18.7	22.2	56.3	21.4	0.118	0.318
	12.7	75.4	12.0	27.4	53.8	18.7	54.1	35.1	10.6	0.807	1.611
	16.3	60.0	23.8	27.4	53.8	18.7	14.7	66.4	18.7	0.576	0.240
mean	30.6	51.6	17.8	27.4	53.8	18.7	32.0	51.0	17.0	0.552	0.360
**Men**											
	25.0	54.5	20.5	27.4	53.8	18.7	19,0	63.2	17.6	0.129	0.306
	27.0	40.3	32.7	27.4	53.8	18.7	19,0	62.1	18.7	0.612	0.737
	20.8	56.1	23.0	27.4	53.8	18.7	17.4	61.1	21.4	0.347	0.185
	21.0	57.0	22.0	27.4	53.8	18.7	27.8	52.7	19.4	0.313	0.312
	6.4	79.4	14.2	27.4	53.8	18.7	54.3	35.6	9.9	1.315	2.234
	10.1	69.0	20.9	27.4	53.8	18.7	23,0	57.1	19.8	0.970	0.781
	23.5	58.4	18.0	27.4	53.8	18.7	15.4	63.2	21.3	0.166	0.449
	51.5	35.0	13.5	27.4	53.8	18.7	37.2	46.1	16.5	0.827	0.463
	51.9	33.1	14.9	27.4	53.8	18.7	56.6	33.7	9.6	0.832	0.403
	30.3	53.8	15.9	27.4	53.8	18.7	37.6	47.4	14.9	0.185	0.259
	22.2	62.0	15.8	27.4	53.8	18.7	27,0	55.4	17.5	0.273	0.224
	26.0	53.7	20.2	27.4	53.8	18.7	24.4	55.7	19.7	0.092	0.071
	46.6	43.2	10.2	27.4	53.8	18.7	47.3	40.2	12.3	0.817	0.188
	16.6	59.0	24.4	27.4	53.8	18.7	21.3	59.6	19,0	0.569	0.352
	21.4	52.5	26.1	27.4	53.8	18.7	20.1	57.5	22.2	0.411	0.179
	26.9	54.4	18.7	27.4	53.8	18.7	37.1	47.8	14.9	0.022	0.415
	19.4	68.7	11.9	27.4	53.8	18.7	17.4	63.2	19.2	0.530	0.467
	21.7	57.6	20.7	27.4	53.8	18.7	13,0	65.3	21.6	0.262	0.487
	11.9	70.0	18.1	27.4	53.8	18.7	14.1	67.7	18,0	0.799	0.153
	17.2	54.4	28.5	27.4	53.8	18.7	16.7	60.4	22.7	0.628	0.235
mean	24.9	55.6	19.5	27.4	53.8	18.7	27.3	54.8	17.9	0.505	0.445

Supplementary table S2.

Percentages of time spent in each task for each worker during the three-day measurement period according to the self-reported diary.

Blue cells mark data for the three workers excluded in the sensitivity analysis

Task:	Check-out	Colonial	FruitVeg	Dairy	Bread	Fresh	Post	Admin	Break
**Women**									
	-	-	-	-	-	87.4	-	-	12.6
	-	-	80.5	-	-	-	-	8.0	11.5
	-	56.2	-	-	-	-	17.3	8.9	17.6
	-	29.0	2.9	-	48.5	-	-	6.6	12.9
	84.5	-	-	-	-	-	-	-	15.5
	67.3	22.5	-	-	-	-	-	-	10.2
	-	43.3	9.0	-	-	6.0	-	32.8	9.0
	91.7	3.3	-	-	-	-	-	-	5.0
	-	-	-	-	-	-	90.0	-	10.0
	-	79.9	-	-	-	-	-	-	20.1
	-	-	-	-	25.5	-	-	60.4	14.2
	-	0.7	-	-	82.4	-	-	-	16.9
	70.2	-	-	-	-	-	21.6	-	8.2
	9.1	78.4	-	-	-	-	-	-	12.5
	80.2	6.4	-	-	-	-	-	2.4	11.0
	-	-	86.7	-	-	-	-	-	13.3
**Men**									
	-	-	81.8	-	-	-	-	7.3	10.9
	-	1.3	-	77.2	-	-	-	5.0	16.5
	-	50.2	4.3	28.2	-	-	2.2	-	15.2
	-	53.8	-	16.3	-	-	-	14.4	15.4
	88.3	6.7	-	-	-	-	-	-	5.0
	17.4	23.3	-	-	-	43.8	-	1.2	14.3
	-	10.9	-	-	-	71.1	-	6.1	11.9
	42.6	43.0	-	-	-	-	-	-	14.4
	90.6	-	-	-	-	-	-	-	9.4
	50.9	-	-	-	-	-	39.7	-	9.4
	-	21.9	48.2	-	-	-	-	15.8	14.0
	-	53.0	16.6	-	-	6.6	-	11.6	12.2
	54.7	-	-	14.1	-	-	8.7	9.8	12.7
	14.6	-	-	27.1	-	4.2	40.8	-	13.3
	-	84.4	-	-	-	-	-	2.6	13.0
	50.6	-	-	-	-	-	40.8	-	8.6
	2.5	13.2	2.8	67.1	-	-	-	2.2	12.2
	-	-	-	-	-	83.8	-	4.1	12.1
	-	-	91.4	-	-	-	-	2.5	6.1
	-	92.9	-	-	-	-	-	-	7.1

Supplementary table S3.

Physical behavior compositions during the four-hour surveilled measurement period in each task for each worker; i.e. the basis of the Task Exposure Matrix

Zeros in green cells were replaced by 0.01%. Blue cells mark data for the three workers excluded in the sensitivity analysis

Task:	Check-out			Colonial			FruitVeg			Dairy			Bread		
	%sitting	%standing	%moving	%sitting	%standing	%moving	%sitting	%standing	%moving	%sitting	%standing	%moving	%sitting	%standing	%moving
**Women**															
	-	-	-	-	-	-	-	-	-	-	-	-	-	-	-
	-	-	-	-	-	-	7.1	73.7	19.2	-	-	-	-	-	-
	-	-	-	21.7	48.4	29.9	-	-	-	-	-	-	-	-	-
	-	-	-	30.7	45.2	24.1	-	-	-	-	-	-	10.2	70.1	19.7
	70.2	23.5	6.3	-	-	-	-	-	-	-	-	-	-	-	-
	63.4	30.5	6.0	-	-	-	-	-	-	-	-	-	-	-	-
	-	-	-	18.8	55.8	25.4	-	-	-	-	-	-	-	-	-
	0.3	68.1	31.5	7.4	66.1	26.5	-	-	-	-	-	-	-	-	-
	-	-	-	13.2	61.7	25.2	-	-	-	-	-	-	-	-	-
	-	-	-	11.9	72.6	15.5	-	-	-	-	-	-	-	-	-
	-	-	-	-	-	-	-	-	-	-	-	-	-	-	-
	-	-	-	-	-	-	-	-	-	-	-	-	5.2	70.6	24.2
	71.3	20.0	8.7	-	-	-	-	-	-	-	-	-	-	-	-
	98.6	1.1	0.3	28.1	61.6	10.4	-	-	-	-	-	-	-	-	-
	0.1	94.5	5.4	10.2	75.4	14.5	-	-	-	-	-	-	-	-	-
**Men**															
	-	-	-	-	-	-	21.1	58.0	20.9	-	-	-	-	-	-
	-	-	-	-	-	-	-	-	-	14.5	49.9	35.6	-	-	-
	-	-	-	16.1	73.7	10.2	-	-	-	11.0	69.4	19.6	-	-	-
	-	-	-	-	-	-	-	-	-	2.5	78.4	19.1	-	-	-
	0.1	76.1	23.8	5.0	62.5	32.6	-	-	-	-	-	-	-	-	-
	-	-	-	12.1	66.2	21.7	-	-	-	-	-	-	-	-	-
	-	-	-	-	-	-	-	-	-	-	-	-	-	-	-
	71.2	20.6	8.2	10.4	64.1	25.4	-	-	-	-	-	-	-	-	-
	94.3	4.2	1.5	5.7	66.0	28.3	-	-	-	-	-	-	-	-	-
	60.2	38.1	1.7	11.1	72.5	16.4	-	-	-	-	-	-	-	-	-
	-	-	-	-	-	-	0.0	86.0	14.0	-	-	-	-	-	-
	-	-	-	12.6	62.2	25.2	-	-	-	-	-	-	-	-	-
	78.5	20.6	0.8	13.3	62.7	24.0	-	-	-	-	-	-	-	-	-
	79.6	9.0	11.4	18.6	59.4	22.0	-	-	-	-	-	-	-	-	-
	-	-	-	5.1	61.3	33.6	-	-	-	-	-	-	-	-	-
	65.6	31.9	2.6	-	-	-	-	-	-	-	-	-	-	-	-
	-	-	-	-	-	-	-	-	-	1.7	86.1	12.2	-	-	-
	-	-	-	-	-	-	-	-	-	13.2	74.1	12.7	-	-	-
	-	-	-	-	-	-	13.3	68.1	18.6	17.2	68.7	14.1	-	-	-
	-	-	-	27.3	50.3	22.4	-	-	-	-	-	-	-	-	-

Supplementary table S3 (continued)

Fresh			Post			Admin			Break		
%sitting	%standing	%moving	%sitting	%standing	%moving	%sitting	%standing	%moving	%sitting	%standing	%moving
1.2	83.7	15.1	-	-	-	-	-	-	-	-	-
-	-	-	-	-	-	94.6	3.8	1.5	-	-	-
-	-	-	37.1	42.1	20.8	-	-	-	-	-	-
-	-	-	-	-	-	-	-	-	-	-	-
-	-	-	-	-	-	-	-	-	0.0	0.0	100.0
-	-	-	-	-	-	-	-	-	-	-	-
-	-	-	-	-	-	-	-	-	1.4	53.1	45.6
-	-	-	-	-	-	-	-	-	74.6	17.5	8.0
-	-	-	-	-	-	-	-	-	53.8	22.8	23.5
-	-	-	-	-	-	-	-	-	71.8	16.7	11.6
-	-	-	-	-	-	48.6	48.0	3.3	69.2	23.6	7.1
-	-	-	-	-	-	-	-	-	15.7	62.7	21.5
-	-	-	-	-	-	-	-	-	39.0	37.7	23.3
-	-	-	-	-	-	-	-	-	33.5	46.1	20.4
-	-	-	-	-	-	-	-	-	54.5	34.6	10.9
-	-	-	-	-	-	-	-	-	46.9	38.5	14.5
-	-	-	-	-	-	-	-	-	0.0	63.4	36.6
-	-	-	0.0	65.2	34.8	-	-	-	0.0	100.0	0.0
-	-	-	-	-	-	96.2	1.5	2.2	0.0	36.2	63.8
-	-	-	-	-	-	-	-	-	77.4	12.8	9.8
4.5	60.3	35.2	-	-	-	-	-	-	0.0	69.3	30.7
3.5	80.9	15.5	-	-	-	-	-	-	-	-	-
-	-	-	-	-	-	-	-	-	57.5	28.4	14.1
-	-	-	-	-	-	-	-	-	40.4	38.9	20.7
-	-	-	-	-	-	-	-	-	85.4	6.9	7.8
-	-	-	-	-	-	76.3	16.6	7.1	21.4	57.9	20.7
13.5	63.4	23.1	-	-	-	93.9	4.3	1.8	57.5	18.8	23.6
-	-	-	-	-	-	-	-	-	68.6	20.5	10.9
-	-	-	2.3	80.5	17.2	-	-	-	16.4	17.9	65.5
-	-	-	-	-	-	71.8	19.7	8.5	71.0	19.4	9.6
-	-	-	1.1	85.5	13.4	-	-	-	77.4	13.6	9.0
-	-	-	-	-	-	-	-	-	76.5	15.9	7.5
5.3	65.2	29.6	-	-	-	-	-	-	44.7	38.0	17.3
4.0	80.4	15.6	-	-	-	-	-	-	55.4	36.8	7.8
-	-	-	-	-	-	-	-	-	53.2	36.8	9.9
